# Evaluation of Human Leukocyte Antigen-A (HLA-A), Other Non-HLA Markers on Chromosome 6p21 and Risk of Nasopharyngeal Carcinoma

**DOI:** 10.1371/journal.pone.0042767

**Published:** 2012-08-07

**Authors:** Wan-Lun Hsu, Ka-Po Tse, Sharon Liang, Yin-Chu Chien, Wen-Hui Su, Kelly J. Yu, Yu-Juen Cheng, Ngan-Ming Tsang, Mow-Ming Hsu, Kai-Ping Chang, I-How Chen, Tzu-I Chen, Czau-Siung Yang, Alisa M. Goldstein, Chien-Jen Chen, Yu-Sun Chang, Allan Hildesheim

**Affiliations:** 1 Genomics Research Center, Academia Sinica, Taipei, Taiwan; 2 Genome Medicine Core, Chang Gung Molecular Medicine Research Center, Chang Gung University, Taoyuan,Taiwan; 3 Division of Cancer Epidemiology and Genetics, National Cancer Institute, Rockville, Maryland, United States of America; 4 Food and Drug Administration, Rockville, Maryland, United States of America; 5 Molecular and Genomic Epidemiology Research Center, China Medical University Hospital, Taichung, Taiwan; 6 Molecular Epidemiology Core, Chang Gung Molecular Medicine Research Center, Chang Gung University, Taoyuan, Taiwan; 7 Department of Biomedical Sciences, Graduate Institute of Biomedical Sciences, Chang Gung University, Taoyuan, Taiwan; 8 Division of Cancer Prevention, National Cancer Institute, Rockville, Maryland, United States of America; 9 Graduate Institute of Epidemiology and Preventive Medicine, College of Public Health, National Taiwan University, Taipei, Taiwan; 10 Department of Radiation Oncology, Chang Gung Memorial Hospital at Lin-Kou, Taoyuan, Taiwan; 11 Department of Otolaryngology, National Taiwan University Hospital and National Taiwan University College of Medicine, Taipei, Taiwan; 12 Department of Otolaryngology, Chang Gung Memorial Hospital at Lin-Kou, Taoyuan, Taiwan; 13 Graduate Institute of Microbiology, College of Medicine, National Taiwan University, Taipei, Taiwan; Johns Hopkins University, United States of America

## Abstract

**Background:**

The association between human leukocyte antigen (*HLA*) genes (located in the Major Histocompatibility Complex [MHC] region of chromosome 6p21) and NPC has been known for some time. Recently, two genome-wide association studies (GWAS) conducted in Taiwan and China confirmed that the strongest evidence for NPC association was mapped to the MHC region. It is still unclear, however, whether these findings reflect direct associations with Human Leukocyte Antigen (*HLA*) genes and/or to other genes in this gene-rich region.

**Methods:**

To better understand genetic associations for NPC within the MHC region of chromosome 6, we conducted an evaluation that pooled two previously conducted NPC case-control studies in Taiwan (N = 591 cases and N = 521 controls). PCR-based genotyping was performed for 12 significant SNPs identified within 6p21 in the Taiwan NPC GWAS and for the *HLA-A* gene (exons 2 and 3).

**Findings:**

After confirming homogeneity between the two studies, pooled odds ratios (OR) and 95% confidence intervals (CI) were estimated by logistic regression. We found that *HLA-A* (p-trend = 0.0006) and rs29232 (within the *GABBR1* gene; p-trend = 0.005) were independent risk factors for NPC after adjustment for age, gender, study and each other. NPC risk was highest among individuals who were homozygous for the HLA-A*0207 risk allele and carriers of the rs29232 risk allele (A).

**Conclusion:**

Our study suggests that most of the SNPs significantly associated with NPC from GWAS reflect previously identified *HLA-A* associations. An independent effect of rs29232 (*GABBR1*), however, remained, suggesting that additional genes within this region might be associated with NPC risk.

## Introduction

While Epstein-Barr virus (EBV) is known to be closely associated with the development of nasopharyngeal carcinoma (NPC) [1]–[3], it is also believed that genetic factors are important determinants of NPC risk [4], [5]. Recently completed genome-wide association studies (GWAS) have identified the strongest evidence for NPC association within the Major Histocompatibility Complex (MHC) region of chromosome 6p21 where the Human Leukocyte Antigen (*HLA*) genes are located [6], [7]. These findings reinforce results from case-control studies conducted since the early 1970s, suggesting an association between specific alleles and/or haplotypes of *HLA* genes and NPC [8]–[10]. For example, the associations between *HLA-A* alleles *0207 (risk allele) and *1101 (protective allele), and *HLA-B* allele *4601 (risk allele in strong linkage disequilibrium with HLA-A*0207) have been consistently reported. These associations are biologically plausible since NPC is closely linked to EBV infection and *HLA* alleles are known to be critical for proper presentation of viral antigens to the immune system. Recently, Yan Li *et al.* genotyped 233 SNPs on chromosome 6p in 360 NPC patients and 360 healthy controls, and has demonstrated that in addition to known *HLA-A* gene, multiple chromosome 6p susceptibility loci contribute to the risk of NPC, possibly through *GABBR1* and *NEDD9* loss of function [11]. However, it remains unclear whether the *HLA* associations observed to date are due to a causal effect by single *HLA* alleles, combinations of *HLA* alleles (haplotypes), or genes in the MHC region that are closely linked to *HLA* alleles/haplotypes. To help clarify this issue, we performed a study that pooled individuals from two previous NPC case-control studies in Taiwan. More specifically, we examined whether the 12 newly reported SNPs within the MHC region identified through GWAS are independent of *HLA* effects or if they simply reflect the previously reported *HLA* Class I associations.

## Materials and Methods

### Study population

Two case-control studies of NPC in Taiwan were included. One was conducted by the National Taiwan University Hospital (NTUH) and Mackay Memorial Hospital (MMH) in collaboration with the U.S. National Cancer Institute [8]. The second was conducted by Chang-Gung University (CGU) and Chang-Gung Memorial Hospital (CGMH) [7]. The first study has previously reported on HLA-NPC associations [8]. The latter study served as the basis for the recently published NPC GWAS from Taiwan [7].

#### NTUH/MMH study

Details of the study have been described elsewhere [12], [13]. Briefly, 378 incident NPC cases that were histologically confirmed and 328 community controls who had no history of NPC were identified between July 15, 1991 and December 31, 1994. NPC cases were restricted to individuals less than 75 years, with no previous diagnosis for NPC and who were residents of Taipei city/county for more than 6 months. One control was selected for each case, individually-matched by sex, age (within 5-years), and residence area (same district or township). Of the 378 eligible cases and 372 eligible controls, 375 cases (99%) and 327 controls (88%) agreed to participate in the study and signed informed consent. Peripheral blood was successfully collected from 369 cases and 320 controls. For this analysis, 347 cases and 294 controls are included; the remaining 22 cases and 26 controls are not included because DNA was exhausted by previous testing for other factors. Institutional Review Boards at the National Taiwan University and the National Cancer Institute in The United States (both for and under the NCI Special Studies IRB) approved the study protocol and informed consent. Written consent was obtained from study participants.

#### CGU/CGMH Study

Blood samples from 244 patients initially diagnosed with NPC in CGMH at Lin-kou (Taoyuan County, Taiwan) were collected. For comparison, 228 healthy local residents of Taoyuan County were recruited through a project designated “Integrated Delivery System of Health Screening, Taoyuan, Taiwan” by CGU, CGMH, and the Health Bureau of Taoyuan County, Taiwan. Control samples were randomly selected within strata to represent the male/female ratio and age distribution of NPC cases. Controls affected by any type of cancer and with a family history of NPC were excluded. All cases and controls were of Han Chinese origin. This study protocol and informed content were reviewed and approved by the Institutional Review Board of Chang Gung Memorial Hospital (CGMH IRB No. 95-0733B & 99-0654C). Written informed consent was obtained from all study participants.

### DNA Extraction, SNP selection and testing, and *HLA* typing

#### NTUH/MMH study

DNA from peripheral blood lymphocytes was extracted by QIAamp Blood Kit (Qiagen, Valencia, CA) following the manufacturer's protocols. We included 12 candidate SNPs that were found to be significantly associated with NPC susceptibility on Chromosome 6p21.3 from the GWAS study conducted in Taiwan [7]. The genotyping of the target SNPs (Table S1) was designed as Assay-by-Design method with a commercially available TaqMan SNP genotyping assay kit and GeneAmp PCR System 9700 (Applied Biosystems, Foster City, CA), according to the manufacturer's instructions. *HLA* typing for *HLA-A* and *HLA-B* was performed as previously described [8]. Briefly, *HLA* Class I A and B typing were performed by PCR-based co-amplification of the polymorphic exon 2 and exon 3 of each locus followed by detection and allele assignment using a reverse line-blot typing system. Relevant to this evaluation, the typing system used could not identify all HLA-A*11** genotypes observable in Asian populations and so HLA-A*11** genotypes were evaluated as a single group (referred henceforth as HLA-A*11**). HLA-A*1101 represents approximately 81% of all HLA-A*11** positive individuals in Taiwan [14].

#### CGU/CGMH Study

DNA from peripheral blood was extracted using a Qiagen DNA isolation kit (Qiagen, Valencia, CA, USA). The quality and concentration of genomic DNA for each sample was determined using a NanoDrop Spectrophotometer (NanoDrop Products, Thermo Scientific, Wilmington, DE). Genome-wide genotyping experiments using Illumina HumanHap550v3_A BeadChips was performed by an Illumina-certified service provider, Genizon Biosciences (Genizon BioSciences, Canada) and results from this effort previously published [7]. *HLA-A* alleles were typed by directly sequencing the region covering exons 2 and 3 of the *HLA-A* gene [15]. All sequences were assembled and compared with the NCBI dbMHC database for identification of the *HLA-A* genotype [16].

### Statistical analysis

**Figure 1:**
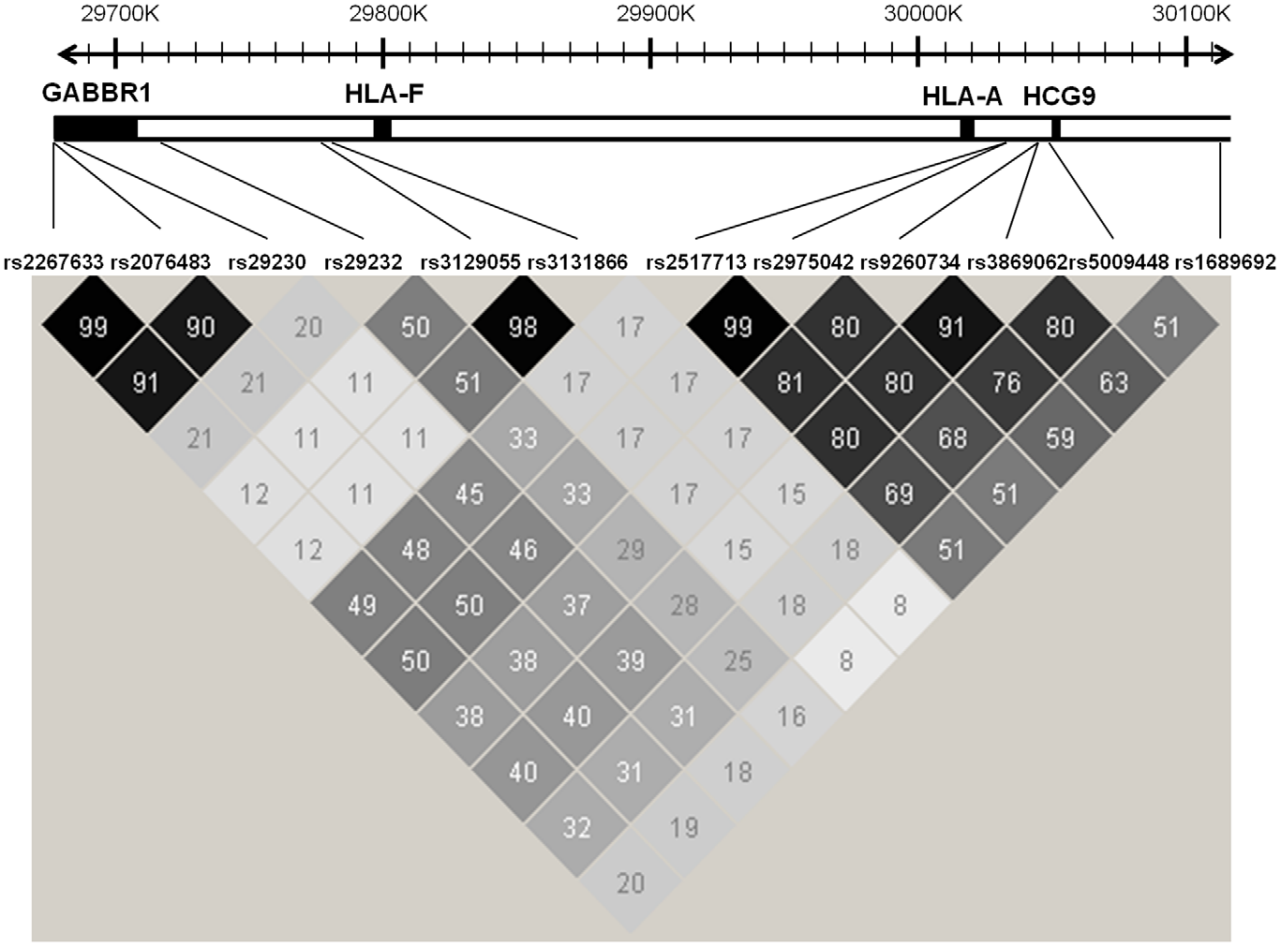
Haploview Map Showing Location and R-squared for 12 Markers Selected for Study.

Allele frequencies were computed among controls and compared between studies using the Pearson's χ^2^ test. Risk of NPC associated with specific SNPs and *HLA* alleles of interest was evaluated by estimating odds ratios (ORs) with 95% confidence intervals (CIs) using unconditional logistic regression that controlled for age, sex, study (i.e., CGU/CGMH or NTUH/MMH) and genetic covariates (e.g. HLA adjusted for SNPs and SNPs adjusted for HLA). The likelihood ratio test was used to evaluate heterogeneity between the two studies. Armitage Trend tests were used to evaluate evidence for trend by including the categorical variable of interest as a continuous variable in the logistic regression model. To reduce the number of comparisons, we used Haploview software to select tag SNPs for further evaluation. Using a threshold of r^2^≥0.8, five tag SNPs were selected: rs2076483, rs29232, rs3129055, rs3869062, rs16896923 (Figure 1). With respect to *HLA*, we focused our evaluation on *HLA-A* alleles that fulfilled the following criteria: 1) allele frequency among controls ≥5% and 2) evidence from the literature for a consistent association with NPC. Using these two criteria, the following *HLA-A* alleles were selected for evaluation: HLA-A*0207 and HLA-A*11**. Since HLA-A*0207 and HLA-A*11** are alleles of the same gene, a single variable with the following categories was constructed for the analysis: 11/11 (most protected group), 11/others, 11/0207, others/others, 0207/others and 0207/0207 (highest risk group). Sensitivity analyses were performed within specific studies, as follows, to ensure the robustness of our main findings: HLA-A*1101 specific analysis within the CGU/CGUH study, HLA-B*4601 analysis within the NTUH/MMH study, and analysis stratified by Chinese ethnicity (Fukienese, which represent the majority of Taiwanese, vs not) within the NTUH/MMH study. All statistical tests were two-sided. Analyses were performed using SAS release 9.1 (SAS Institute, Cary, NC).

## Results

The mean age for the cases and controls was 45.8 (SD = 11.6) and 45.3 (SD = 11.8) years, respectively in the NTUH/MMH study and 48.1 (SD = 11.9) and 47.4 (SD = 10.8) years, respectively in the CGU/CGMH study. Both studies were comprised primarily of males (69.4% in the NTUH/MMH study and 72.0% in the CGU/CGMH study).

Comparable allelic frequencies were observed among controls from the two studies for the 12 studied SNPs and *HLA-A* alleles (A*0207 * A*11) of interest (p-heterogeneity >0.05 for all comparisons –Table S2). Risk estimates for these markers were also comparable between the two studies (p-heterogeneity >0.05 –Table S3). Given that allelic frequencies and risk estimates were comparable across studies, pooled results are reported herein. Furthermore, to reduce the number of comparisons, analyses focused on the five tag SNPs identified in our study (rs2076483, rs29232, rs3129055, rs3869062, rs16896923; see Methods and Figure 1) and the two *HLA-A* alleles of interest (HLA-A*0207 and HLA-A*11). Results for the 5 tag SNPs of interest are presented in Table 1. After adjustment for age, gender, study, and *HLA-A*, a significant association remained for a single SNP located within *GABBR1* (rs29232) (p-trend = 0.005). Results for the *HLA-A* alleles of interest are presented in Table 2. As shown in the table, after adjustment for age, gender, study, and rs29232, a significant association remained for *HLA-A* (p-trend = 0.0006). Consistent with the adjusted models presented in Tables 1 and 2, when the joint effect of rs29232 and *HLA-A* was evaluated, we observed an association between rs29232 and NPC within strata of *HLA-A* and an association between *HLA-A* and NPC within strata of rs29232 (Table S4). Of interest, although limited by small numbers, we noted that individuals who were homozygous for HLA-A*0207 and who carried a risk allele of rs29232, had a 29-fold increase in risk of NPC (95% CI = 6.2–135) when compared to those who were homozygous for HLA-A*11** and who did not carry a risk allele of rs29232 allele.

**Table 1:** Pooled Distribution, Odds Ratios and 95% Confidence Intervals for the Association Between Chromosome 6p21 TagSNPs and NPC.

SNP	Genotype	Case (%)	Control (%)	OR (95% CI)^1^	OR (95% CI)^2^
***GABBR1***					
rs2076483					
	AA	403 (68.8)	273 (52.4)	1	1
	AG	166 (28.3)	213 (40.9)	0.52 (0.40–0.68)	0.72 (0.51–1.0)
	GG	17 (2.9)	35 (6.7)	0.32 (0.18–0.59)	0.71 (0.33–1.5)
	P- Trend			<0.0001	0.07
rs29232					
	GG	114 (19.4)	171 (33.1)	1	1
	AG	290 (49.5)	259 (50.1)	1.7 (1.3–2.3)	1.3 (0.91–1.8)
	AA	182 (31.1)	87 (16.8)	3.2 (2.2–4.5)	1.9 (1.2–2.9)
	P- Trend			<0.0001	0.005
***HLA-F***					
rs3129055					
	AA	222 (37.8)	246 (47.3)	1	1
	AG	260 (44.2)	231 (44.4)	1.2 (0.96–1.6)	0.90 (0.67–1.2)
	GG	106 (18.0)	43 (8.3)	2.8 (1.9–4.2)	1.4 (0.90–2.3)
	P- Trend			<0.0001	0.43
***HCG9***					
rs3869062					
	AA	343 (60.0)	223 (44.0)	1	1
	AG	207 (36.2)	232 (45.8)	0.58 (0.45–0.74)	0.67 (0.36–1.2)
	GG	22 (3.9)	52 (10.3)	0.28 (0.17–0.47)	1.0 (0.28–3.5)
	P- Trend			<0.0001	0.46
rs16896923					
	AA	413 (71.0)	292 (56.8)	1	1
	AG	163 (28.0)	195 (37.9)	0.59 (0.46–0.77)	0.80 (0.55–1.2)
	GG	6 (1.0)	27 (5.3)	0.16 (0.07–0.40)	0.36 (0.13–1.0)
	P- Trend			<0.0001	0.07

1 adjusted for age, gender, study (study = CGU/CGMH or NTUH/MMH).

2 adjusted for age, gender, study, HLA-A.

**Table 2:** Pooled Distribution, Odds Ratios and 95% Confidence Intervals for the Association Between Chromosome 6p21 HLA-A Alleles and NPC.

HLA-A	Case (%)	Control (%)	OR (95% CI)^1^	OR (95% CI)^2^
11/11	27 ( 4.6)	65 (12.5)	1	1
11/Others	195 (33.0)	220 (42.2)	2.1 (1.3–3.4)	1.9 (1.1–3.2)
11/0207	31 ( 5.3)	16 ( 3.1)	4.4 (2.1–9.4)	3.6 (1.6–8.1)
Others/Others	211 (35.7)	163 (31.3)	3.1 (1.9–5.1)	2.3 (1.3–4.0)
0207/Others	104 (17.6)	55 (10.6)	4.5 (2.6–7.9)	2.9 (1.5–5.6)
0207/0207	23 ( 3.9)	2 ( 0.4)	30 (6.5–135)	17 (3.6–83)
P-Trend			<0.0001	0.0006

1 adjusted for age, gender, study.

2 adjusted for age, gender, study, rs29232.

Sensitivity analyses that evaluated HLA-A*1101 within the CGU/CGMH study and that controlled for *HLA-B* alleles within the NTUH/MMH study yielded results that are comparable to those reported overall (data not shown). There was also no significant evidence for heterogeneity by Chinese ethnicity within the NTUH/MMH study (p-heterogenetity = 0.81 for *HLA-A* and 0.06 for rs29232), although the effect of rs29232 appeared weaker among Chinese of Fukienese descent (OR = 1.2 for those homozygous for risk allele) than among Chinese of non-Fukienese descent (OR = 6.3 for those homozygous for risk allele) (Table S5).

## Discussion

In this study, we evaluated SNPs within the MHC region of chromosome 6p21 that were recently reported to be significantly associated with NPC from a GWAS conducted in Taiwan by one of our groups (CGU/CGMH). By pooling two NPC case-control studies conducted in Taiwan, we were interested in evaluating whether the significant SNPs identified by the GWAS were reflective of or independent from previously reported *HLA* Class I-NPC associations. Of the 12 SNPs considered, all but one appeared to reflect previously reported associations between HLA-A*0207 (risk allele), *HLA*-11** (protective allele) and NPC. Only rs29232, a SNP located within the *GABBR1* gene remained significant after adjustment for *HLA-A* effects, suggesting that it may represent a genetic determinant that is independent from the HLA-A*0207/*HLA*-11** associations.

Dissecting gene disease associations within the MHC region has been particularly difficult because it is a gene-rich region. This is further complicated by the fact that the *HLA* genes are highly polymorphic and that extended linkage disequilibrium (LD) exists between genes within this region. A report by Yan Li *et al.* has suggested that multiple loci within 6p21.3 were associated with NPC susceptibility [11]. In their study, they've confirmed the association between *HLA-A/GABBR1* genes and risk of NPC by genotyping 233 SNPs on chromosome 6p in 360 NPC patients and 360 healthy controls. Furthermore, using the genotyping data and quantitative real-time PCR, they've identified several micro-deleted regions covering *NEDD9* and *GABBR1* genes and demonstrated that reduced expression of both genes in NPC tumor cells when compared to adjacent normal tissues. Since these genes are closely linked to the significant loci we validated in this study, we also checked the expression of *GABBR1* and *NEDD9* mRNA in our microarray dataset [17]. As the data shown in Table S6, we found that there is no significant difference in the expressions of these two genes in NPC and adjacent non-tumor tissues. We also reviewed the expression data from our previously conducted study on 31 NPC tissues and 10 normal controls and found similar results (http://www.ncbi.nlm.nih.gov/geo/query/acc.cgi?acc=GSE12452) [18]. In addition, we examined the mRNA expression levels of *NEDD9* and *GABBR1* in 10 matched-pairs NPC and adjacent non-tumor tissues by quantitative real-time PCR (Figure S1 and Table S7). Similar to the findings of our microarray analyses, there is no significant altered expression of these two genes in our clinical biopsies (p = 0.233 and 0.261 for *NEDD9* and *GABBR1*, respectively; paired t-test). Since the sample size is relatively small, further validation is warranted.

Our results, while confirming the association between *HLA-A* and NPC and suggesting the possible involvement of an additional gene in the region, *GABBR1*, should be interpreted with caution. First, although we pooled two studies to conduct our evaluation, our sample size remained modest given the tight LD patterns observed in the MHC region. We attempted to limit concerns about multiple comparisons by restricting our evaluation to the 5 SNPs that tagged the 12 SNPs reported from the Taiwan GWAS and by focusing on the *HLA-A* alleles for which the strongest evidence for association exists. Second, our study was not designed to define specific functional implications of the *HLA-A* or *GABBR1* associations. While there is ample biological plausibility for the association between HLA*0207, HLA*11** and NPC, including evidence that HLA-A*1101 is efficient at presenting EBV antigens to the immune system [19], we cannot determine whether observed associations are driven by these two specific alleles or by alleles in other *HLA* genes in tight LD with HLA-A. Of note, however, two recent reports that evaluated *HLA* Class I association with NPC reported that the association between the *HLA-B* allele for which the most long-standing evidence for association exists (HLA-B*4601) was only observed in the presence of HLA-A*0207, and that the effect of HLA-A*0207 was independent of the presence of HLA-B*4601 [10], [20]. Third, the association observed between *GABBR1* and NPC, while not significantly different by whether or not individuals were of Fukienese vs other Chinese descent, there was suggestive evidence that the effects were stronger for individuals who were not of Fukienese descent suggesting caution in interpreting our finding for an independent effect for rs29232 within *GABBR1*.

In summary, we report here that most of the associations identified in a recent GWAS of NPC within the MHC region on 6p21 are likely to reflect previously reported *HLA* Class I associations with NPC. An independent association between rs29232 (*GABBR1*) and NPC cannot be ruled out based on our results.

## Supporting Information

Table S1 **SNP IDs and Corresponding Assay Name/ID for the 12 SNPs Used for TaqMan Genotyping.**
(DOCX)

Table S2 **Distribution of SNPs and HLA-A Alleles of Interest Among Controls, by Study.**
(DOCX)

Table S3 **Odds Ratios and 95% Confidence Intervals for the Association Between Chromosome 6p21 SNPs and NPC, by Study and Overall.**
(DOCX)

Table S4 **Stratified Analysis of HLA-A and GABBR1 SNP rs29232.**
(DOCX)

Table S5 **Odds Ratios and 95% Confidence Intervals for the Association Between rs29232 and HLA-A and NPC, by Chinese Ethnicity within the NTUH/MMH Study.**
(DOCX)

Table S6 **Microarray Results of** ***NEDD9*** **and** ***GABBR1*** **Transcripts Expression in NPC Biopsies.**
(DOCX)

Table S7 **Primer list for Quantative Real-time RT-PCR.**
(DOCX)

Figure S1 **The Expression of** ***NEDD9*** **and** ***GABBR1*** **mRNA in NPC Biopsy Tissues.** The Changes in the Expression of *NEDD9* (A) and *GABBR1* (B) mRNA in 10 Matched-pairs of NPC and Adjacent Normal Tissues were Analyzed by Quantitative Real-time RT-PCR as Described in Methods. 18s rRNA was Used as Internal Control, and the Sequence of the Primers Used was Showed in Supplementary Primer List (Table S7).
(TIF)
