# Supplementary material for: Evaluation of Human Leukocyte Antigen-A (HLA-A), Other Non-HLA Markers on Chromosome 6p21 and Risk of Nasopharyngeal Carcinoma
Source: PLoS One. 2012 Aug 7;7(8):e42767. doi: 10.1371/journal.pone.0042767 (PMC3413673; doi:10.1371/journal.pone.0042767)
Supplement: Table S1 — SNP IDs and Corresponding Assay Name/ID for the 12 SNPs Used for TaqMan Genotyping. (DOCX) [file pone.0042767.s001.docx]

Table S1. SNP IDs and Corresponding Assay Name/ID for the 12 SNPs Used for TaqMan Genotyping

| SNP ID | Assay Name/ID |
| --- | --- |
| rs2076483 | V_112607-1-001 |
| rs2975042 | V_112607-2-002 |
| rs3129055 | C__27465238_10 |
| rs2267633 | C__16200758_10 |
| rs16896923 | C__33415129_10 |
| rs3131866^+^ | C__29847681_10 |
| rs2517713 | C__26546328_10 |
| rs5009448 | C__26544301_10 |
| rs3869062 | C__27527150_10 |
| rs9260734 | C__29630815_10 |
| rs29232 | C___8943238_30 |
| rs29230 | C____596215_1_ |

^+^ rs9258122 has merged into [rs3131866](http://www.ncbi.nlm.nih.gov/projects/SNP/snp_ref.cgi?rs=3131866)
