# Supplementary material for: Evaluation of Human Leukocyte Antigen-A (HLA-A), Other Non-HLA Markers on Chromosome 6p21 and Risk of Nasopharyngeal Carcinoma
Source: PLoS One. 2012 Aug 7;7(8):e42767. doi: 10.1371/journal.pone.0042767 (PMC3413673; doi:10.1371/journal.pone.0042767)
Supplement: Table S2 — Distribution of SNPs and HLA-A Alleles of Interest Among Controls, by Study. (DOCX) [file pone.0042767.s002.docx]

Table S2. Distribution of SNPs and *HLA-A* Alleles of Interest Among Controls, by Study

|  |  |  | CGU/CGMH | NTUH/MMH | Heterogeneity |
| --- | --- | --- | --- | --- | --- |
| Gene | SNPs | Minor Allele | Control (%) | Control (%) | p-value |
| *GABBR1* | rs2267633 | G | 25 | 28.4 | 0.23 |
|  | rs2076483 | G | 25 | 28.8 | 0.17 |
|  | rs29230 | G | 25 | 24.7 | 0.92 |
|  | rs29232 | A | 42.8 | 41.2 | 0.61 |
| *HLA-F* | rs3129055 | G | 31.1 | 30 | 0.68 |
|  | rs3131866^+^ | A | 31.1 | 30.1 | 0.71 |
| *HLA-A* | rs2517713 | C | 37.3 | 37.9 | 0.85 |
|  | rs2975042 | C | 37 | 38.1 | 0.7 |
|  | rs9260734 | A | 33.9 | 35 | 0.73 |
|  | rs3869062 | G | 33 | 33.3 | 0.92 |
|  |  | 0207 | 7.2 | 7.2 | 0.97 |
|  |  | 11** | 34.9 | 35.3 | 0.88 |
| *HCG9* | rs5009448 | A | 37.5 | 37.4 | 0.98 |
|  | rs16896923 | G | 25.2 | 23.4 | 0.51 |

^+^ rs9258122 has merged into [rs3131866](http://www.ncbi.nlm.nih.gov/projects/SNP/snp_ref.cgi?rs=3131866)
