# Supplementary material for: Evaluation of Human Leukocyte Antigen-A (HLA-A), Other Non-HLA Markers on Chromosome 6p21 and Risk of Nasopharyngeal Carcinoma
Source: PLoS One. 2012 Aug 7;7(8):e42767. doi: 10.1371/journal.pone.0042767 (PMC3413673; doi:10.1371/journal.pone.0042767)
Supplement: Table S3 — Odds Ratios and 95% Confidence Intervals for the Association Between Chromosome 6p21 SNPs and NPC, by Study and Overall. (DOCX) [file pone.0042767.s003.docx]

Table S3. Odds Ratios and 95% Confidence Intervals for the Association Between Chromosome 6p21 SNPs and NPC, by Study and Overall

| SNPs | Genotype | CGU/CGMH | NTUH/MMH | Study Heterogeneity | Pooled Data |
| --- | --- | --- | --- | --- | --- |
|  |  | OR (95% CI)^1^ | OR (95% CI)^1^ |  | OR (95% CI) ^2^ |
| ***GABBR1*** | | | | | |
| rs2267633 | AA | 1 | 1 | 0.81 | 1 |
|  | AG | 0.47 (0.31-0.71) | 0.56 (0.40-0.79) |  | 0.52 (0.41-0.68) |
|  | GG | 0.31 (0.11-0.84) | 0.39 (0.19-0.79) |  | 0.36 (0.20-0.64) |
|  | P-Trend^3^ | <0.0001 | <0.0001 |  | <0.0001 |
| rs2076483 | AA | 1 | 1 | 0.84 | 1 |
|  | AG | 0.47 (0.31-0.71) | 0.56 (0.40-0.78) |  | 0.52 (0.40-0.68) |
|  | GG | 0.31 (0.11-0.84) | 0.33 (0.15-0.70) |  | 0.32 (0.18-0.59) |
|  | P-Trend^3^ | <0.0001 | <0.0001 |  | <0.0001 |
| rs29230 | AA | 1 | 1 | 0.77 | 1 |
|  | AG | 0.47 (0.31-0.71) | 0.56 (0.40-0.77) |  | 0.52 (0.41-0.67) |
|  | GG | 0.31 (0.11-0.84) | 0.48 (0.15-1.5) |  | 0.38 (0.18-0.80) |
|  | P-Trend^3^ | <0.0001 | 0.0004 |  | <0.0001 |
| rs29232 | GG | 1 | 1 | 0.39 | 1 |
|  | AG | 1.6 (1.0-2.6) | 1.7 (1.2-2.5) |  | 1.7 (1.3-2.3) |
|  | AA | 4.0 (2.3-6.9) | 2.7 (1.7-4.4) |  | 3.2 (2.2-4.5) |
|  | P-Trend^3^ | <0.0001 | <0.0001 |  | <0.0001 |
| ***HLA-F*** | | | | | |
| rs3129055 | AA | 1 | 1 | 0.07 | 1 |
|  | AG | 1.1 (0.74-1.6) | 1.4 (1.0-1.9) |  | 1.2 (0.96-1.6) |
|  | GG | 4.6 (2.4-8.8) | 2.0 (1.2-3.4) |  | 2.8 (1.9-4.2) |
|  | P-Trend^3^ | <0.0001 | 0.004 |  | <0.0001 |
| rs3131866 | GG | 1 | 1 | 0.07 | 1 |
|  | AG | 1.1 (0.74-1.6) | 1.4 (0.98-1.9) |  | 1.2 (0.95-1.6) |
|  | AA | 4.6 (2.4-8.8) | 2.0 (1.2-3.4) |  | 2.8 (1.9-4.2) |
|  | P-Trend^3^ | <0.0001 | 0.005 |  | <0.0001 |
| ***HLA-A*** | | | | | |
| rs2517713 | AA | 1 | 1 | 0.36 | 1 |
|  | AC | 0.46 (0.31-0.68) | 0.68 (0.48-0.95) |  | 0.58 (0.45-0.75) |
|  | CC | 0.24 (0.12-0.49) | 0.27 (0.15-0.49) |  | 0.26 (0.16-0.40) |
|  | P-Trend^3^ | <0.0001 | <0.0001 |  | <0.0001 |
| rs2975042 | AA | 1 | 1 | 0.47 | 1 |
|  | AC | 0.47 (0.31-0.69) | 0.65 (0.47-0.91) |  | 0.57 (0.45-0.74) |
|  | CC | 0.23 (0.11-0.47) | 0.24 (0.13-0.44) |  | 0.23 (0.15-0.37) |
|  | P-Trend^3^ | <0.0001 | <0.0001 |  | <0.0001 |
| rs9260734 | GG | 1 | 1 | 0.57 | 1 |
|  | AG | 0.43 (0.29-0.64) | 0.57 (0.41-0.80) |  | 0.51 (0.40-0.66) |
|  | AA | 0.29 (0.14-0.63) | 0.28 (0.14-0.54) |  | 0.28 (0.17-0.47) |
|  | P-Trend^3^ | <0.0001 | <0.0001 |  | <0.0001 |
| rs3869062 | AA | 1 | 1 | 0.39 | 1 |
|  | AG | 0.46 (0.31-0.68) | 0.67 (0.48-0.93) |  | 0.58 (0.45-0.74) |
|  | GG | 0.28 (0.13-0.61) | 0.28 (0.14-0.58) |  | 0.28 (0.17-0.47) |
|  | P-Trend^3^ | <0.0001 | 0.0002 |  | <0.0001 |
| HLA-A*11^4^ | No/No | 1 | 1 | 0.25 | 1 |
|  | No/Yes | 0.47 (0.32-0.70) | 0.73 (0.53-1.0) |  | 0.62 (0.48-0.79) |
|  | Yes/Yes | 0.27 (0.13-0.57) | 0.27 (0.14-0.51) |  | 0.27 (0.17-0.44) |
|  | P-Trend^3^ | <0.0001 | 0.0001 |  | <0.0001 |
| HLA-A*0207 | No/No | 1 | 1 | 0.99 | 1 |
|  | No/Yes | 1.9 (1.2-3.1) | 2.0 (1.3-3.1) |  | 2.0 (1.4-2.7) |
|  | Yes/Yes | ∞ | 4.6 (0.97-21) |  | 13 (3.0-55) |
|  | P-Trend^3^ | <0.0001 | 0.0002 |  | <0.0001 |
| ***HCG9*** | | | | | |
| rs5009448 | GG | 1 | 1 | 0.16 | 1 |
|  | AG | 0.47 (0.32-0.70) | 0.57 (0.41-0.79) |  | 0.53 (0.41-0.68) |
|  | AA | 0.38 (0.20-0.73) | 0.44 (0.25-0.77) |  | 0.41 (0.27-0.63) |
|  | P-Trend^3^ | <0.0001 | 0.0002 |  | <0.0001 |
| rs16896923 | AA | 1 | 1 | 0.87 | 1 |
|  | AG | 0.57 (0.38-0.85) | 0.61 (0.43-0.85) |  | 0.59 (0.46-0.77) |
|  | GG | 0.20 (0.06-0.60) | 0.13 (0.03-0.57) |  | 0.16 (0.07-0.40) |
|  | P-Trend^3^ | 0.0001 | 0.0001 |  | <0.0001 |

^1^ Adjusted for age and gender

^2^ Adjusted for age, gender, study

^3^ Based on additive model

^4^ HLA-A*11 includes 1101, 1102, 1104, 1119, 1126, 1136, 1139, and 1140
