# Supplementary material for: Evaluation of Human Leukocyte Antigen-A (HLA-A), Other Non-HLA Markers on Chromosome 6p21 and Risk of Nasopharyngeal Carcinoma
Source: PLoS One. 2012 Aug 7;7(8):e42767. doi: 10.1371/journal.pone.0042767 (PMC3413673; doi:10.1371/journal.pone.0042767)
Supplement: Table S4 — Stratified Analysis of HLA-A and GABBR1 SNP rs29232. (DOCX) [file pone.0042767.s004.docx]

Table S4. Stratified Analysis of HLA-A and GABBR1 SNP rs29232

| *HLA-A*combination | rs29232 | Case (%) | Control (%) | OR (95% CI) |
| --- | --- | --- | --- | --- |
| 11/11 | GG | 22 (3.8) | 59 (11.4) | 1 |
|  | AG | 4 (0.7) | 5 (1.0) | 2.2 (0.55-9.1) |
|  | AA | 0 | 0 | - |
| 11/other | GG | 65 (11.1) | 86 (16.6) | 2.0 (1.1-3.6) |
|  | AG | 119 (20.3) | 128 (24.8) | 2.5 (1.4-4.3) |
|  | AA | 8 (1.4) | 3 (0.6) | 7.5 (1.8-31) |
| 11/0207 | GG | 1 (0.2) | 0 | ∞ |
|  | AG | 29 (5.0) | 14 (2.7) | 5.3 (2.4-12) |
|  | AA | 1 (0.2) | 2 (0.4) | 1.2 (0.10-14) |
| Other/other | GG | 25 (4.3) | 26 (5.0) | 2.5 (1.2-5.3) |
|  | AG | 104 (17.8) | 85 (16.4) | 3.3 (1.8-5.7) |
|  | AA | 81 (13.8) | 52 (10.1) | 4.2 (2.3-7.6) |
| 0207/other | GG | 1 (0.2) | 0 | ∞ |
|  | AG | 31 (5.3) | 27 (5.2) | 3.1 (1.5-6.3) |
|  | AA | 72 (12.3) | 28 (5.4) | 6.8 (3.5-13) |
| 0207/0207 | GG | 0 | 0 | - |
|  | AG | 3 (0.5) | 0 | ∞ |
|  | AA | 20 (3.4) | 2 (0.4) | 29 (6.2-135) |

Adjusted for age, gender and study
