# Supplementary material for: Evaluation of Human Leukocyte Antigen-A (HLA-A), Other Non-HLA Markers on Chromosome 6p21 and Risk of Nasopharyngeal Carcinoma
Source: PLoS One. 2012 Aug 7;7(8):e42767. doi: 10.1371/journal.pone.0042767 (PMC3413673; doi:10.1371/journal.pone.0042767)
Supplement: Table S5 — Odds Ratios and 95% Confidence Intervals for the Association Between rs29232 and HLA-A and NPC, by Chinese Ethnicity within the NTUH/MMH Study. (DOCX) [file pone.0042767.s005.docx]

Table S5. Odds Ratios and 95% Confidence Intervals for the Association Between rs29232 and *HLA-A* and NPC, by Chinese Ethnicity within the NTUH/MMH Study

| SNP/  Gene | Genotype | Fukienese | | Non-Fukienese | | p-value for Hetero-  geneity |
| --- | --- | --- | --- | --- | --- | --- |
|  |  | Number of Cases/Controls | OR (95% CI)^1^ | Number of Cases/Controls | OR (95% CI) ^1^ |  |
| rs29232 | GG | 59/68 | 1 | 12/31 | 1 | 0.06 |
|  | AG | 149/96 | 1.3 (0.80-2.1) | 29/46 | 1.2 (0.45-3.2) |  |
|  | AA | 75/41 | 1.2 (0.64-2.3) | 18/7 | 6.3 (1.5-26) |  |
|  | p-Trend^2^ |  | 0.51 |  | 0.02 |  |
|  |  |  |  |  |  |  |
| *HLA-A* | 11/11 | 12/26 | 1 | 3/11 | 1 | 0.81 |
|  | 11/Others | 99/81 | 2.61 (1.17-5.83) | 23/38 | 1.97 (0.41-9.35) |  |
|  | 11/0207 | 20/10 | 3.71 (1.22-11.30) | 5/4 | 4.20 (0.51-34.77) |  |
|  | Others/others | 107/72 | 2.92 (1.25-6.82) | 16/25 | 1.29 (0.23-7.13) |  |
|  | 0207/others | 39/17 | 4.49 (1.60-12.60) | 14/17 | 2.83 (0.41-19.69) |  |
|  | 0207/0207 | 9/2 | 9.08 (1.52-54.32) | - | - |  |
|  | p-Trend^2^ |  | 0.02 |  | 0.81 |  |

^1^ Adjusted for age, gender and HLA-A/rs29232

^2^ Based on additive model
