# Supplementary material for: Evaluation of Human Leukocyte Antigen-A (HLA-A), Other Non-HLA Markers on Chromosome 6p21 and Risk of Nasopharyngeal Carcinoma
Source: PLoS One. 2012 Aug 7;7(8):e42767. doi: 10.1371/journal.pone.0042767 (PMC3413673; doi:10.1371/journal.pone.0042767)
Supplement: Table S6 — Microarray Results of NEDD9 and GABBR1 Transcripts Expression in NPC Biopsies. (DOCX) [file pone.0042767.s006.docx]

Table S6. Microarray Results of *NEDD9* and *GABBR1* Transcripts Expression in NPC Biopsies

| Gene Symbol | Probe ID | Average of Fold Change (T/N) |
| --- | --- | --- |
| *NEDD9* | 202149_at | 0.617 (0.439 – 1.193) |
|  | 202150_s | 0.962 (0.528 – 1.334) |
|  | 233223_at | 1.256 (0.55 – 1.532) |
| *GABBR1* | 204316_s_at | 0.952 (0.473 – 1.607) |
|  | 238569_at | 0.662 (0.662 – 1.109) |
| ^a^Probe ID: from Human Genome U133 Plus 2.0 Array (Affymetrix, Santa Clara, CA, USA). | | |
