# Supplementary material for: Evaluation of Human Leukocyte Antigen-A (HLA-A), Other Non-HLA Markers on Chromosome 6p21 and Risk of Nasopharyngeal Carcinoma
Source: PLoS One. 2012 Aug 7;7(8):e42767. doi: 10.1371/journal.pone.0042767 (PMC3413673; doi:10.1371/journal.pone.0042767)
Supplement: Table S7 — Primer list for Quantative Real-time RT-PCR. (DOCX) [file pone.0042767.s007.docx]

Table S7. Primer List for Quantative Real-time RT-PCR

| **Gene Name** | **Forward Primer (5’ to 3’)** | **Reverse Primer (5’ to 3’)** |
| --- | --- | --- |
| *NEDD9* | CTACAGGGTAAGGAGGAGTTT | TGGGTCTCACATTGGTCAT |
| *GABBR1* | GCTGGGAAGAACATGCTATC | CTTCAAGCCAGGTACGAACT |
| *18S rRNA* | CGAGCCGCCTGGATACC | CCTCAGTTCCGAAAACCAACAA |
