# Supplementary figures and images for: Evaluation of Human Leukocyte Antigen-A (HLA-A), Other Non-HLA Markers on Chromosome 6p21 and Risk of Nasopharyngeal Carcinoma
Source: PLoS One. 2012 Aug 7;7(8):e42767. doi: 10.1371/journal.pone.0042767 (PMC3413673; doi:10.1371/journal.pone.0042767)

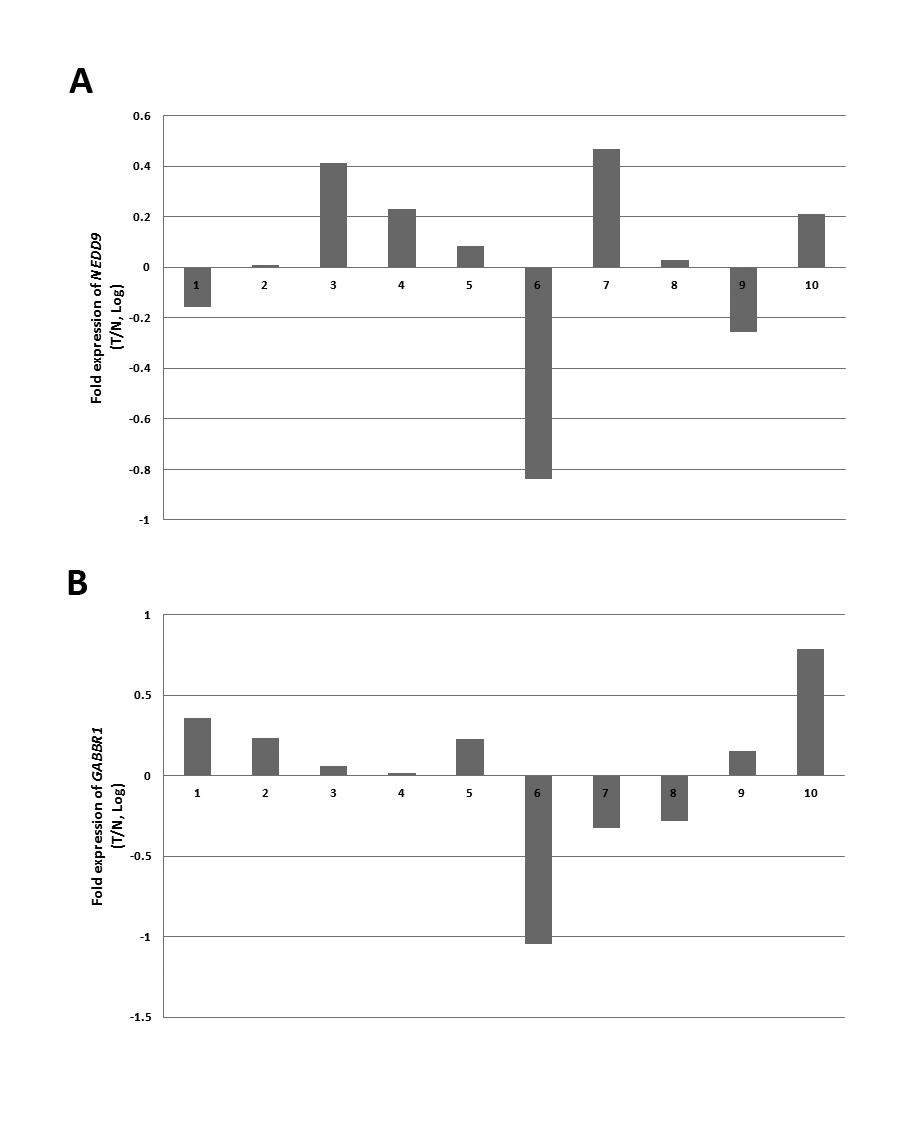

Supplement: Figure S1 — The Expression of NEDD9 and GABBR1 mRNA in NPC Biopsy Tissues. The Changes in the Expression of NEDD9 (A) and GABBR1 (B) mRNA in 10 Matched-pairs of NPC and Adjacent Normal Tissues were Analyzed by Quantitative Real-time RT-PCR as Described in Methods. 18s rRNA was Used as Internal Control, and the Sequence of the Primers Used was Showed in Supplementary Primer List (Table S7). (TIF) [file pone.0042767.s008.tif]
